# Probiotic Potential of *Pediococcus pentosaceus* M6 Isolated from Equines and Its Alleviating Effect on DSS-Induced Colitis in Mice

**DOI:** 10.3390/microorganisms13050957

**Published:** 2025-04-22

**Authors:** Jialong Cao, Jianqiang Zhang, Hui Wu, Yanan Lin, Xinlan Fang, Siqin Yun, Ming Du, Shaofeng Su, Yuanyi Liu, Na Wang, Tugeqin Bao, Dongyi Bai, Yiping Zhao

**Affiliations:** 1 Key Laboratory of Equus Germplasm Innovation, Ministry of Agriculture and Rural Affairs, Hohhot 010018, China; 17647314067@163.com (J.C.); zhjianqiang123@126.com (J.Z.); 13474980304@163.com (H.W.); linyanan@emails.imau.edu.cn (Y.L.); fangxinlan201@163.com (X.F.); 15924516522@163.com (S.Y.); duming@imau.edu.cn (M.D.); 13470105913@163.com (Y.L.); 15804814519@163.com (N.W.); tvgqin@gmail.com (T.B.); baidongyi1983@163.com (D.B.); 2 Inner Mongolia Key Laboratory of Equine Science Research and Technology Innovation, Inner Mongolia Agricultural University, Hohhot 010018, China; 3 Equus Research Center, Inner Mongolia Agricultural University, Hohhot 010018, China; 4 College of Animal Science, Inner Mongolia Agricultural University, Hohhot 010018, China; 5 Inner Mongolia Academy of Agricultural & Animal Husbandry Sciences, Hohhot 010031, China; sushaofeng2020@163.com

**Keywords:** equine, inflammatory bowel disease, intestinal contents, *Pediococcus pentosaceus*, 16S rDNA

## Abstract

Colitis in equines has high morbidity and mortality rates, which severely affects the development of the equine-breeding industry. With the issuance of antibiotic bans, there is an urgent need for healthier and more effective alternatives. In recent years, probiotics have been widely used as microbial feed additives in animal husbandry, playing a crucial role in preventing and treating diarrhea and regulating host immune function. In this study, we isolated and screened a strain with rapid and stable acid production using bromocresol purple, litmus milk coloration tests, and acid production performance assessments. Based on morphological characteristics, physiological and biochemical properties, and 16S rDNA identification, the strain was identified as *Pediococcus pentosaceus* and named M6. The *Pediococcus pentosaceus* M6 exhibited stable growth and tolerance to high temperatures, acid and bile salt concentrations, and simulated gastrointestinal fluid environments. The M6 strain demonstrated good antibacterial effects against *Escherichia coli*, *Staphylococcus aureus*, and *Salmonella*. The M6 strain did not produce hemolysis zones on Columbia blood agar plates, indicating its high safety, and was found to be insensitive to 12 antibiotics, including cephalexin and neomycin. Additionally, intervention in mice with dextran sulfate sodium (DSS)-induced colitis alleviated weight loss and shortened colon length. To a certain extent, it regulated the expression of inflammatory cytokines and the gut microbiota within the body and reduced inflammatory cell infiltration and intestinal barrier damage. In summary, the isolated *Pediococcus pentosaceus* M6 strain exhibited excellent probiotic properties and could alleviate DSS-induced colitis in mice, suggesting its potential application value as a probiotic in animal husbandry.

## 1. Introduction

Inflammatory bowel disease (IBD) is a gastrointestinal disorder with an unknown etiology that poses a significant challenge to both humans and animals and is often difficult to treat effectively [1,2]. Despite the availability of various medications for IBD management, their therapeutic outcomes remain suboptimal. This condition not only represents a major health concern for humans, but also frequently affects equine species, particularly in the form of colitis, enteritis, and proctitis, which have emerged as prevalent diseases threatening the well-being of horses in recent years [3]. Both bacterial agents (such as *Salmonella typhimurium*, *Clostridium perfringens*, *Clostridium difficile*, and *Clostridium piliforme*) and viruses (primarily equine rotavirus and equine coronavirus) can trigger colitis in equines, with diarrhea or hemorrhagic diarrhea being the common clinical manifestation across various etiologies, ultimately leading to disruption of the gut microbiota balance [4,5]. Antibiotics are the primary therapeutic approach for managing colitis in horses. However, their efficacy is often limited, and significant side effects persist, hence, the urgency to identify alternative and more efficacious substitutes has intensified.

Lactic acid bacteria (LAB) are a group of bacteria that produce lactic acid through the fermentation of sugars. They primarily include genera such as *Lactobacillus*, *Bifidobacterium*, and *Streptococcus*. These bacteria naturally exist in food, the environment, and the intestines of animals [6,7]. When consumed in adequate amounts, lactic acid bacteria that confer health benefits to the host are referred to as probiotics [8]. Probiotics are a rich source of bioactive compounds with numerous functions and potential clinical applications [9,10]. They improve the intestinal barrier function by modulating cytokine production, inducing regulatory T cells, enhancing microbial killing and intestinal cell survival, regulating the gut microbiota composition, and ameliorating abnormal mucosal immune responses associated with intestinal inflammation [11]. *Pediococcus pentosaceus* is a Gram-positive bacterium of the Streptococcaceae family, belonging to the genus *Pediococcus* [12]. As a probiotic, *Pediococcus pentosaceus* exhibits various health benefits, including anti-inflammatory, antioxidant, and cholesterol-lowering effects, enhancement of the intestinal barrier function, and improvement in the gut microbiota diversity [13,14]. Furthermore, research has shown that *Lactobacillus plantarum* A3 isolated from healthy horses has the potential to treat ulcerative colitis in mice by modulating the composition and structure of the intestinal microbiota [15].

This study aimed to isolate probiotics from healthy Mongolian horses, evaluate their biological functions and probiotic effects, and investigate their mitigating effects on dextran sulfate sodium (DSS)-induced ulcerative colitis in a mouse model. This endeavor sought to uncover potential therapeutic drugs and strategies for managing IBD.

## 2. Materials and Methods

### 2.1. Screening and Identification of Equine-Derived Strains

#### 2.1.1. Primary Screening

The intestinal contents of 10 Mongolian horses (5 females and 5 males), aged 2~3 years, at Salaqi Horse Farm, Baotou City, Inner Mongolia Autonomous Region, People’s Republic of China (PRC), were analyzed using sterile centrifuge tubes. All the horses were certified as healthy by veterinary examination and free from digestive system diseases. The samples were transported at low temperature back to the laboratory at Inner Mongolia Agricultural University, Hohhot, China. There, 4 mL of the Mongolian horse intestinal content were transferred into a test tube containing 36 mL of a phosphate-buffered saline (PBS) solution and mixed thoroughly using a shaker. Then, 4 mL of the mixed sample were aspirated into another test tube containing 36 mL of PBS, repeating this process to create six dilution gradients of 10^−1^, 10^−2^, 10^−3^, 10^−4^, 10^−5^, and 10^−6^ [16]. After that, 200 μL of the sample dilutions from three dilution gradients (10^−4^, 10^−5^, 10^−6^) were dispensed onto the prepared MRS agar using a pipette (Qingdao Hi-tech Industrial Park Hope Bio-technology Co., Ltd., Qingdao, China). The samples were evenly spread using a sterile spreader. Once the samples were set up, they were inverted and incubated at 37 °C in a constant-temperature anaerobic workstation for 24~36 h. After anaerobic incubation, an inoculating loop was used to select individual colonies and purify them on an MRS agar medium by anaerobic incubation at 37 °C for 24 h. Inside an aseptic bench, the purified and propagated strains were streaked again on an MRS agar medium and anaerobically incubated for 24 h. The streaking process was repeated twice to further purify the colonies, and the strains that had undergone multiple purifications were transferred to an MRS liquid medium and anaerobically incubated for 24 h. Upon completion, 80% sterile glycerol was added to the bacterial suspension in a 3:1 volume ratio, mixed well, and stored at −80 °C for future experiments with appropriate labeling. The strains stored at −80 °C were later activated. De Man, Rogosa, and Sharpe (MRS) agar containing bromocresol purple was prepared. The candidate strains were activated and spread on MRS agar plates containing bromocresol purple. The strains were incubated under anaerobic conditions at 37 °C for 24 h. The strains that turned the purple MRS agar yellow (indicating acid production) were selected for further experimentation.

#### 2.1.2. Re-Screening

The strains preserved at −80 °C were activated. Following the instructions, litmus milk medium (Qingdao Hi-tech Industrial Park Hope Bio-technology Co., Ltd., Qingdao, China) was prepared and dispensed into 96-well plates. The activated strains were introduced into the litmus milk medium at a volume ratio of 1:10 and incubated anaerobically for 24 h. After incubation, the state of the litmus milk medium was observed. Owing to the strong acid-producing capability of the strains, the medium exhibited a pink curd-like appearance. The strains that exhibited this pink curd-like appearance were selected for further experimentation. The superior strains identified through the litmus milk assay were activated and purified through one subculture. These strains were inoculated into an MRS liquid medium at 1% inoculum size and incubated anaerobically at 37 °C for 24 h. The pH of the bacterial suspensions was measured at 0, 2, 4, 6, 8, 12, 16, and 24 h. Using incubation time as the horizontal axis and pH as the vertical axis, a time–pH curve was generated by plotting incubation time (x-axis) against pH values (y-axis) to evaluate acid production kinetics. The strain with the best acid-producing performance was selected for Gram staining, followed by microscopic observation and physiological and biochemical characterization.

### 2.2. DNA Extraction and 16S rDNA-Based Gene Amplification

The purified strains preserved through activation and purchased standard strains were cultured in 5 mL of MRS broth for 24~38 h. Genomic DNA was extracted from these cultures using a Tiangen Bacterial Genomic DNA Extraction Kit (TIANGEN BIOTECH (BEIJING) Co., Ltd., Beijing, China). Polymerase chain reaction amplification was performed using universal primers specific to bacterial 16S rDNA, with the upstream primer 27F (sequence: 5′-AGAGTTTGATCCTGGCTCA-3′) and the downstream primer 1492R (sequence: 5′-GGTTACCTTGTTACGACTT-3′). The polymerase chain reaction products were analyzed by means of agarose gel electrophoresis. The PCR conditions were set as follows: initial denaturation at 94 °C for 5 min, followed by 30 cycles of denaturation at 94 °C for 30 s, annealing at 57 °C for 45 s, extension at 72 °C for 2 min, final extension at 72 °C for 10 min, and storage at 4 °C. Five microliters of the PCR product were loaded onto a prepared 1.2% agarose gel containing 2 μL/mL of nucleic acid stain for electrophoresis. The results were obtained using a UV gel imaging system. After electrophoresis, clear bands were excised and subjected to gel extraction. The recovered DNA samples were then sent to Shanghai Sangon Biotech (Co., Ltd., Shanghai, China) for sequencing to obtain 16S rDNA sequences of the strains. The obtained 16S rDNA sequences were analyzed for homology using the BLAST 1.4.0 tool available online at NCBI (https://www.ncbi.nlm.nih.gov accessed on 25 February 2025). Bacterial 16S rDNA sequences with high homology to the query sequences were selected, and a phylogenetic tree was constructed using MEGA6.0 software.

### 2.3. Determination of Growth Performance of Pediococcus pentosaceus M6

The selected strains were purified and cultured anaerobically at 37 °C for 48 h in MRS broth. The cultured strains were then inoculated into a fresh MRS broth (1% *v*/*v* inoculum). Bacterial growth was monitored by measuring optical density at 600 nm (OD600) using a microplate reader (BioTek Instruments, Inc., Winooski, VT, USA) at 0, 2, 4, 6, 8, 12, 16, 24, 32, and 48 h post-inoculation. A growth curve was generated by plotting culture time (x-axis) against OD600 nm values (y-axis) to characterize the growth kinetics of the studied strains.

### 2.4. Determination of Heat Resistance, Acid Resistance, and Bile Salt Resistance of Pediococcus pentosaceus M6

#### 2.4.1. Determination of Heat Resistance

The strains were inoculated into MRS broth, thoroughly mixed by vortexing, and then subjected to heat treatment in a water bath at different temperatures (37 °C, 45 °C, 55 °C, 65 °C, and 75 °C) for 10 min. Following heat exposure, the samples were immediately cooled on ice. Using 37 °C as the control condition, bacterial viability was assessed by measuring optical density at 600 nm (OD600) with a microplate reader. 

#### 2.4.2. Determination of Acid Resistance

A bacterial suspension was prepared by selecting a representative colony from the MRS agar plate. A line was drawn to separate the colony for purification and subsequent culture. The selected colony was inoculated into MRS broth and incubated at 37 °C for 24 h. After incubation, the culture was centrifuged at 4000 rpm for 10 min. The resulting precipitate was washed with PBS, and the 600 nm (OD600) was adjusted to 0.5 ± 0.05. The adjusted bacterial suspension was inoculated (10%, 150 μL inoculum per 1.5 mL medium) into MRS broth adjusted to different pH values (2.0, 3.0, and 4.0) using HCl. The cultures were incubated anaerobically at 37 °C for 48 h. Bacterial growth was monitored by measuring 600 nm (OD600) at 0, 2, 4, 6, 8, 12, 16, 24, 32, and 48 h using a microplate reader. Data were recorded and analyzed to determine acid tolerance characteristics.

#### 2.4.3. Determination of Bile Salt Resistance

To prepare the bacterial suspension, a single typical colony was selected from the MRS agar plate, and line-drawing purification was performed. This colony was inoculated into MRS broth and incubated at 37 °C for 24 h. After the incubation period, the culture was centrifuged at 4000 rpm for 10 min. The resulting precipitate was washed with PBS, and the 600 nm (OD600) was adjusted to a range of 0.5 ± 0.05. The prepared suspension was taken and added to the culture medium with a 10% bacterial dosage. The concentrations of bile salts varied between 0.2%, 0.3%, and 0.4%. The cultures were incubated at 37 °C for 48 h, and 600 nm (OD600) was measured at 0, 2, 4, 6, 8, 12, 16, 24, 32, and 48 h. The collected data were recorded and analyzed.

### 2.5. Test of Simulated Artificial Gastric Juice, Intestinal Juice, and Self-Agglutination Ability

#### 2.5.1. Simulated Artificial Gastric Juice Test

First, 1 mL of the bacterial suspension to be tested was added to 9 mL of sterile artificial gastric juice. This mixture was inoculated into another 9 mL of sterile artificial gastric juice and cultured for 2 h at 37 °C and 150 rpm. After the incubation, a second inoculation into a colony plate counting solution was performed, and it was cultured for 3 h at 37 °C and 100 rpm. The viable bacteria were counted using the colony plate counting method at 0 and 3 h, respectively, and the survival rate was calculated as follows:

Survival rate = colony number at determination time/colony number at 0 × 100%

#### 2.5.2. Simulated Artificial Intestinal Juice Test

First, 1 mL of the bacterial suspension to be tested was added to 9 mL of sterile artificial intestinal juice. This mixture was inoculated into another 9 mL of sterile artificial intestinal juice, and it was cultured for 2 h at 37 °C and 150 rpm. After the incubation, a second inoculation into a colony plate counting solution was performed, and it was cultured for 3 h at 37 °C and 100 rpm. The viable bacteria were counted using the colony plate counting method at 0 and 3 h, respectively, and the survival rate was calculated as follows:

Survival rate = colony number at determination time/colony number at 0 × 100%

#### 2.5.3. Self-Agglutination Ability Test

The candidate strains were activated, cultured at 37 °C for 24 h, centrifuged at 10,000 rpm for 5 min, washed with sterile PBS 3 times, and resuspended with PBS; the 600 nm (OD600) (0.5 ± 0.05) of the bacterial suspension was adjusted and the initial OD value (A_0_) was recorded; 4 mL of the suspension were transferred, put in a test tube, and allowed to stand undisturbed for 24 h; them, the 600 nm (OD600) of the supernatant of the suspension was measured, and the auto-aggregation percentage was calculated as follows [17]:

A% = [(A_0_ − A_t_)/A_0_] × 100%

### 2.6. Evaluation of the Probiotic Effect of Pediococcus pentosaceus M6

#### 2.6.1. Hemolytic Test

Standby strains were selected; 200 μL of the isolated strain solution were evenly spread after 24 h of activation culture on Colombian blood plates (Changde Beekman Biological Co., Changde, China), and an anaerobic culture was conducted at 37 °C for 24 h to observe whether there was hemolysis. *Staphylococcus aureus* was used as the control.

#### 2.6.2. Antimicrobial Activity Determination

The antibacterial activity of the strains was determined by means of the Oxford Cup agar diffusion method. The activated target strains were cultured anaerobically for 24 h, centrifuged at 10,000 rpm for 5 min, and the supernatant was separated and stored in a refrigerator at 4 °C. Then, 200 μL of a fresh bacterial liquid of *Salmonella*, *Escherichia coli*, and *Staphylococcus aureus* was spread evenly on MH agar. After the bacterial liquid was completely absorbed, three Oxford cups were placed on each plate, and 200 μL of the strain supernatant were added to each well. Culture in an incubator was conducted at 37 °C for 24 h, observing whether an inhibition zone was formed and measuring the diameter [18].

#### 2.6.3. Antibiotic Sensitivity Test

After 24 h of activation culture, 200 μL of the isolated strain liquid were evenly spread on an MRS agar plate, and the paper disk diffusion method was used. The strains were tested for penicillin, oxacillin, amikacin, carbenicillin, ampicillin, Polyangiumycin, piperacillin, cefalexin, cefazolin, cefuroxime, ceftazidime, ceftriaxone, cefoperazone, kanamycin, norfloxacin, neomycin, vancomycin, tetracycline, doxycycline, minocycline, gentamicin, doxycycline, and doxycycline [19].

### 2.7. Therapeutic Effect of Pediococcus pentosaceus M6 on DSS-Induced Colitis in Mice

Twenty-four 7-week-old male C57BL/6J mice (SPF grade, Spefo Biotechnology Co., Ltd., Beijing, China) were randomly divided into three groups: a blank control group (BC), a DSS + sterile saline group (DN), and a DSS + M6 group (DM), with 8 mice in each group. Prior to the experiment, the mice were acclimatized for 7 days, with free access to food and water. In the BC group, mice received sterile saline (0.4 mL/d) from day 1~21. In the DN group, mice received DSS (3%) from day 1~7, followed by sterile saline (0.4 mL/d) from day 8~21. The DM group received DSS (3%) from day 1~7 and then a bacterial suspension of M6 at a concentration of 1.0 × 10^9^ CFU/mL (0.4 mL/d) from day 8~21. After the experiment was completed, the mice were fasted for 8–12 h, euthanized, and blood samples were collected via orbital puncture. The mice were then dissected to collect colon tissue and intestinal content samples.

#### 2.7.1. Analysis of Clinical Indexes

During the experiment, the mental status and physical appearance, weight, and fecal characteristics of the mice were recorded every day. The disease activity index (DAI) of the mice was calculated according to the percentage of weight loss, fecal score, and fecal occult blood, and the DAI was recognized as one of the typical indices to evaluate the pathological degree of colitis. The score range of each parameter is 0~4, and the final score is the sum of the parameters. See Table S1 for the specific scoring details.

#### 2.7.2. Histopathological Analysis

First, 1 cm of mouse colon tissue was fixed in 4% paraformaldehyde for 24 h. The tissue was gently rinsed with PBS washes, then dehydrated through an ethanol gradient. After dehydration, the tissue was embedded in paraffin. The embedded tissue was sectioned into 4 μm slices, and the sections were dried in a 60 °C constant-temperature oven. After baking, the sections were stained with hematoxylin and eosin (H&E). The stained paraffin sections were coverslipped with neutral balsam and observed under an optical microscope. The histopathological scoring is shown in Table S2.

#### 2.7.3. Detection of Inflammatory Cytokines in Serum

Mouse blood was centrifuged at 8000 rpm for 15 min, then the serum supernatant was collected for ELISA detection. After that, 50 μL of the test sample and 100 μL of biotinylated antibodies were added to each well. The plate was covered with sealing film and incubated at 37 °C in a light-protected water bath for 60 min. After incubation, the liquid was discarded, and the plate was washed 5 times with wash buffer. Then, 100 μL of HRP-conjugated avidin were added to each well, and incubation was conducted at 37 °C in a water bath protected from light for 20 min. Next, 100 μL of the TMB substrate solution were added to each well, and incubation was conducted at 37 °C in a light-protected water bath for 15 min. Finally, 50 μL of the stop solution were added to each well, and the OD values were measured at 450 nm. The standard curve was used to determine the concentrations of interleukin 1β (IL-1β), interleukin 6 (IL-6), and interleukin 10 (IL-10) in the samples.

#### 2.7.4. Gene Expression Analysis

Total RNA was extracted from colon tissue using the Trizol method, and then reverse-transcribed into cDNA using a reverse transcription kit (TaKaRa Bio Inc., Dalian, China) (Table S3). With *GAPDH* as the internal reference gene, gene expression was detected by means of real-time quantitative PCR (Bio-Rad, Hercules, CA, USA). The relative gene expression levels were calculated using the 2^−∆∆Ct^ method (Tables S4 and S5).

### 2.8. Gut Microbiota Determination

We analyzed the gut microbiome of the mice by first extracting bacterial DNA from colon content samples using an E.Z.N.A. RStool DNA kit and verifying DNA quality with agarose gel electrophoresis. We then amplified the V3–V4 region of the 16S rRNA gene using specific primers (341F/805R) through PCR, prepared sequencing libraries following Illumina protocols, and performed high-throughput sequencing on the NovaSeq 6000 platform (lc-bio Technology Co., Ltd., Hangzhou, China). After sequencing, we processed the data by demultiplexing samples using their barcodes and conducted comprehensive bioinformatic analyses to characterize the microbial community composition in mouse intestines.

### 2.9. Statistics and Analysis of Data

The data were statistically analyzed using Microsoft Excel, and graphs were created using GraphPad Prism 9 software. One-way ANOVA was performed using SPSS 22.0 software, and post-hoc multiple comparisons were performed using the LSD method, with results expressed as the means ± standard deviation, where * indicates a significant difference at *p* < 0.05, and ** indicates a highly significant difference at *p* < 0.01.

## 3. Results

### 3.1. Screening and Identification of Equine-Derived Strains

Lactic acid bacteria can cause acidification (yellow halos) of the bromocresol purple MRS agar medium, as shown in Figure S1A. From these, 96 strains that demonstrated the ability to show distinct yellow halos were selected, purified, cultured, and preserved for further experiments. In the litmus milk test, litmus has a color range from red at pH 4.5 to blue at pH 8.3. When prepared as a medium with a pH of 6.8, it appears in a bluish-purple color. The results of this test are shown in Figure S1B. Ten strains with the most pronounced acidification (pink curdling), designated M1–M10, were selected for the measurement of acid production performance. The acid production curves of these 10 strains are shown in Figure S1C. All 10 lactic acid bacteria showed a rapid decrease in pH during the first 0~8 h of fermentation and reached a stable state after 16 h. Among them, strain M6 showed the best acid production, while strains M1 and M9 showed the worst acid production performance. The acid production curve shows that strain M6 had the best and most stable acid production performance. Therefore, strain M6 was selected for further experiments.

**Figure 1:**
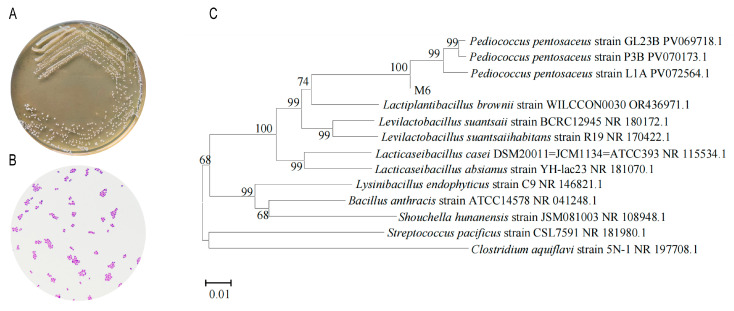
Results of screening and identification of equine-derived strains. (**A**) Purification of the M6 strain; (**B**) microscopic examination of the M6 strain; (**C**) phylogenetic tree of the M6 strain.

Morphologically, the colonies were circular, white, with a moist and smooth surface, arranged singly, in pairs or in short chains, large on MRS agar (Figure 1A). After Gram staining, the bacteria were microscopically revealed to have coccus morphology (Figure 1B). We identified the M6 strain as Gram-positive cocci.

The results of the physiological and biochemical identification of the M6 strain are shown in Table 1. Strain M6 can ferment cellobiose, maltose, mannitol, salicin, sucrose, raffinose, lactose, but not heptuloside, sorbitol, inulin, and glucose; it can gasify catalase, reduce nitrate solution, hydrolyze amylase, be motile, and react with indole, resulting in the formation of an indole ring. It can first be determined that the M6 strain is a *Lactococcus lactis* strain.

The PCR products were electrophoresed on a 1.2% agarose gel and showed clear and single bands. The size of the amplified fragment of the M6 gene was 1500 bp (Figure S1D), which was consistent with the expected design. The homology analysis showed that the homology between strain M6 and *Pediococcus pentosaceus* was 100% (Figure 1C). Based on the morphological, physiological, and biochemical characteristics of the M6 strain and the results of 16S rDNA sequence analysis, the M6 strain was finally identified as *Pediococcus pentosaceus* M6.

### 3.2. Growth Curve and Heat, Acid, and Bile Salt Resistance of Pediococcus pentosaceus M6

**Figure 2:**
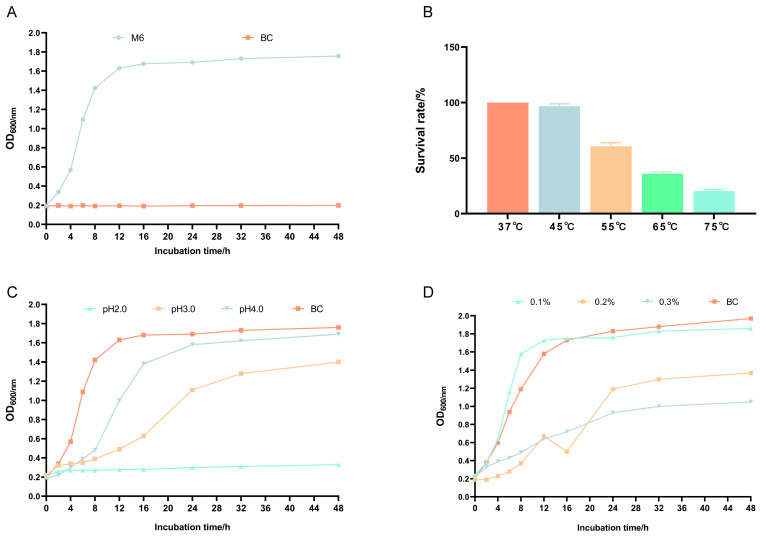
Growth curves and heat, acid, and bile salt resistance of *Pediococcus pentosaceus* M6. (**A**) Growth curve; (**B**) heat resistance; (**C**) acid resistance; (**D**) bile salt resistance.

From Figure 2A, it can be seen that the 600 nm (OD600) value changed little in the 2 h lag phase of *Pediococcus pentosaceus* M6; the 600 nm (OD600) increased almost linearly from 2 to 16 h, which corresponded to the logarithmic growth period of *Pediococcus pentosaceus* M6; after 24 h of incubation, *Pediococcus pentosaceus* M6 entered the stabilization phase, and the 600 nm (OD600) did not decrease much after 30 h, indicating that *Pediococcus pentosaceus* M6 was stable.

The results showed that the survival rate of *Pediococcus pentosaceus* M6 was still more than 60% at 45 °C and 55 °C for 10 min, and more than 20% at 65 °C and 75 °C, indicating that there were detectable viable cells at these temperatures and that *Pediococcus pentosaceus* M6 could survive at high temperatures (Figure 2B). Figure 2C demonstrated results under the acidic conditions of pH 2.0, pH 3.0, and pH 4.0, with a medium with pH 5.8 as the control. In a medium with pH 2.0, the growth of *Pediococcus pentosaceus* M6 stopped or stagnated; in a medium with pH 3.0, the growth was retarded, and in a medium with pH 4.0, it was essentially unaffected and comparable to the control. As shown in Figure 2D, growth under the conditions of 0.1%, 0.2%, and 0.3% bile salts was slow. Compared to the blank control, the growth of *Pediococcus pentosaceus* M6 was not inhibited at 0.1% bile salts, while it was inhibited and slowed at 0.2% and 0.3% bile salts.

### 3.3. Self-Agglutination Ability and Tolerance to Gastric and Intestinal Fluids of Pediococcus pentosaceus M6

Lactic acid bacterial agglutination ability is generally categorized as low, medium, or high, with 16~35% being low self-agglutination ability, 35~50%—medium self-agglutination ability, and more than 50%—high self-agglutination ability. The self-agglutination ability of *Pediococcus pentosaceus* M6 was 61.04 ± 0.02%, indicating that it has a high self-agglutination ability. Table 2 shows the survival of lactic acid bacteria in simulated gastric and intestinal fluids, where the survival rate was 8.99 ± 0.47% for 3 h in the gastric fluid and 4.77 ± 0.58% for 3 h in the intestinal fluid.

### 3.4. Hemolytic Properties of Pediococcus pentosaceus M6

**Figure 3:**
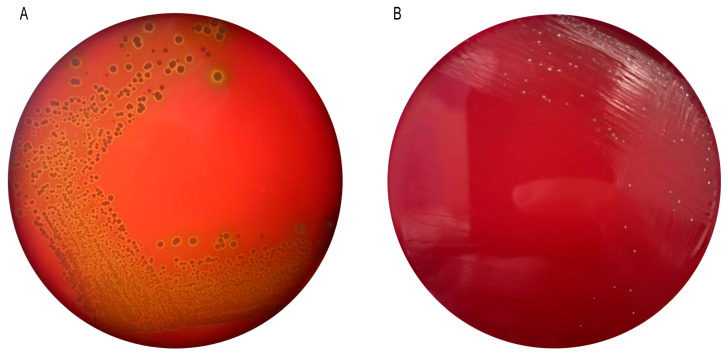
Results of hemolytic properties of strains. (**A**) *Staphylococcus aureus*; (**B**) *Pediococcus pentosaceus* M6.

The results of the hemolysis test of *Pediococcus pentosaceus* M6 are shown in Figure 3. *Staphylococcus aureus* produces a hemolytic ring, and *Pediococcus pentosaceus* M6 does not produce a hemolytic ring, indicating that *Pediococcus pentosaceus* M6 is a safe strain.

### 3.5. Bacteriostatic Properties of Pediococcus pentosaceus M6

The experimental results show that *Pediococcus pentosaceus* M6 had a significant antimicrobial activity on *Escherichia coli*, *Salmonella*, *Staphylococcus aureus*, with the strongest inhibitory effect on *Salmonella*, with the largest diameter of the inhibitory circle of 16.71 ± 0.67 mm; the inhibitory effect on *Staphylococcus aureus* was the second, the diameter of the inhibitory circle being 14.29 ± 0.69 mm; finally, the diameter of the inhibitory circle for *Escherichia coli* was 9.50 ± 0.69 mm (Table 3).

### 3.6. Antibiotic Susceptibility of Pediococcus pentosaceus M6

The sensitivity of *Pediococcus pentosaceus* M6 to 30 antibiotics is variable. It does not produce an inhibitory circle to 12 types of drugs, such as cefadroxil, ceftazidime, butylcarbamazine, gentamicin, kanamycin, neomycin, minocycline, norfloxacin fluoride, norfloxacin, ciprofloxacin, vancomycin, doxycycline, and cotrimoxazole, which means that *Pediococcus pentosaceus* M6 is not sensitive to these 12 types of drugs; it is weakly sensitive to 4 types of drugs, such as cephradine; it is strongly sensitive to 9 types of antibiotics, such as ampicillin; sensitivity to 5 types of antibiotics, such as penicillin, was strongest (Table 4).

### 3.7. Analysis of the Therapeutic Results of Pediococcus pentosaceus M6 in DSS-Induced Colitis

#### 3.7.1. Analysis of Results of Clinical Indices

**Figure 4:**
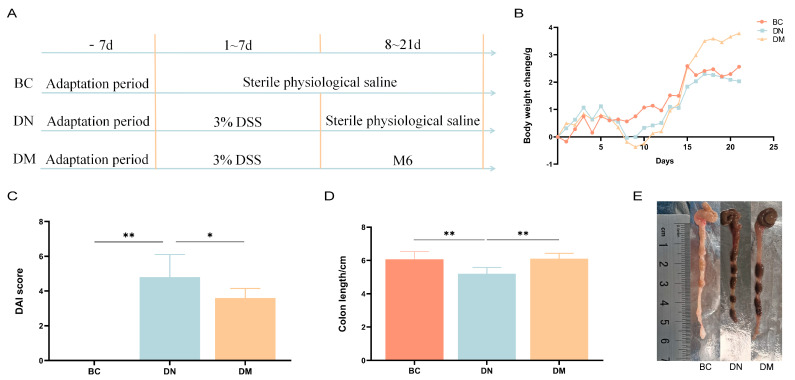
Analysis of the results of clinical indices. Note: * indicates *p* < 0.05; ** indicates *p* < 0.01. (**A**) Experimental process for M6-intervened DSS-induced ulcerative colitis in mice; (**B**) changes in the body weight of the mice; (**C**) DAI scores; (**D**,**E**) length of the colon in each group of mice.

The results of the study showed that the body weight of the mice in the DM group caught up with that of the BC group after 15 days and exceeded that of the BC group after 16 d and beyond, as shown in Figure 4B. This shows that *Pediococcus pentosaceus* M6 can alleviate weight loss in mice with DSS-induced colitis. Disease activity index values are shown in Figure 4C, with a trend toward lower DAI values and a highly significant shortening of colon length in the DM group compared to the DC group (*p* < 0.05) (Figure 4D,E).

#### 3.7.2. Histopathological Observations

**Figure 5:**
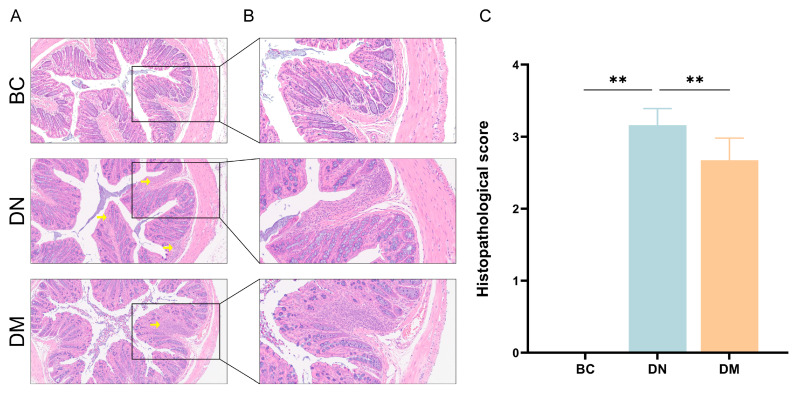
Histopathologic results of the colon of mice in each group. Note: ** indicates *p* < 0.01, yellow arrows indicate inflammatory cell infiltration. (**A**) H&E results of colonic tissues in each group under 100×; (**B**) H&E results of colonic tissues in each group under 200×; (**C**) histopathologic scores of the colon of mice in each group.

The results are shown in Figure 5. Compared with the BC group, the crypts and glands in the DN group were partially dissolved and collapsed, with a large number of inflammatory cells infiltrating. Compared with the DN group, the colonic tissues in the DM group were less damaged, and the extent of inflammatory cell infiltration was small.

#### 3.7.3. Serum Inflammatory Cytokine Levels

**Figure 6:**
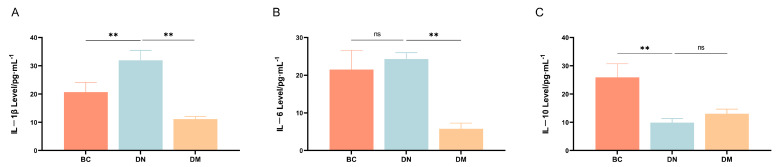
Inflammatory cytokine expression in the serum of each group. Note: ^ns^ indicates *p* ≥ 0.05; ** indicates *p* < 0.01. (**A**) IL−1β; (**B**) IL−6; (**C**) IL-10.

Compared with the BC group, the DN group had very significantly increased levels of the pro-inflammatory factor IL-1β (*p* < 0.01) and non-significantly increased levels of IL-6 (*p* ≥ 0.05), as well as a very significantly reduced expression of the anti-inflammatory factor IL-10 (*p* < 0.01); compared with the DN group, the DM group had very significantly reduced levels of the pro-inflammatory factors IL-6 and IL-1β (*p* < 0.01) and increased levels of the anti-inflammatory factor IL-10 (*p* ≥ 0.05) (Figure 6).

#### 3.7.4. Gene Expression Results

**Figure 7:**
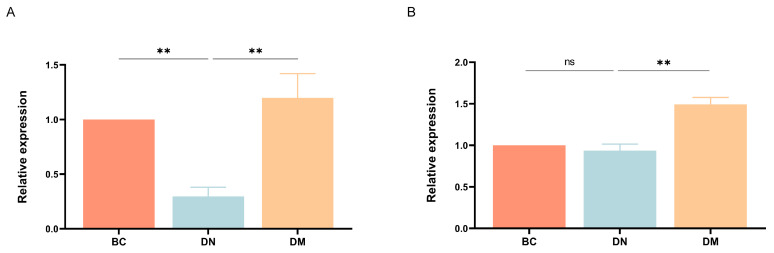
Results of tight junction protein gene expression in the mouse colon tissue by group. Note: ^ns^ indicates *p* ≥ 0.05; ** indicates *p* < 0.01. (**A**) *ZO-1*; (**B**) *occludin*.

The results are shown in Figure 7. Compared with the BC group, the expression of *ZO-1* and *occludin* mRNA was decreased in the DN group; compared with the DN group, the expression of both *ZO-1* and *occludin* mRNA was very significantly higher in the DM group (*p* < 0.01).

### 3.8. Effect of Pediococcus pentosaceus M6 on the Intestinal Flora of Mice with Ulcerative Colitis

#### 3.8.1. Analysis of Fecal Flora Diversity

**Figure 8:**
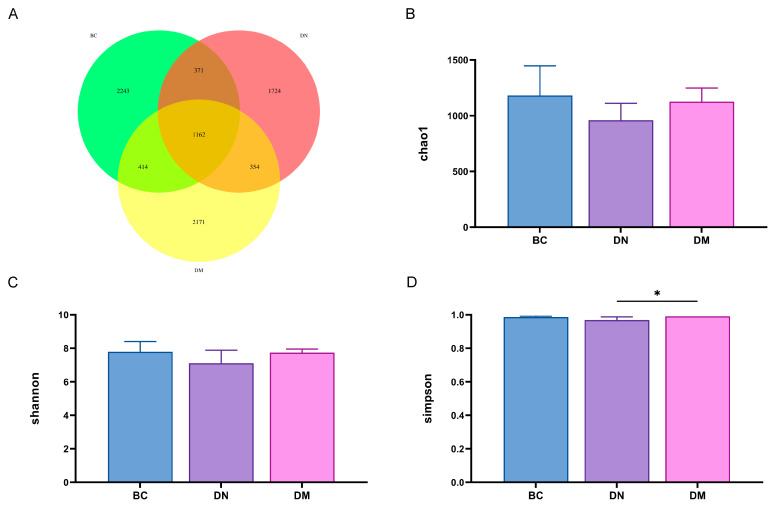
Effect of *Pediococcus pentosaceus* M6 on the diversity of intestinal flora in the mice with DSS-induced colitis. Note: * indicates *p* < 0.05. (**A**) Venn diagram; (**B**) Chao 1 index; (**C**) Shannon’s index; (**D**) Simpson’s index.

The Venn diagram (Figure 8A) shows that the number of OTUs common to the 3 groups was 1162, the number of OTUs common to the BC and DN groups was 371, the number of OTUs common to the BC and DM groups was 414, and the number of OTUs common to the DN and DM groups was 354. Compared with the BC group, the Chao 1 index, Shannon’s index, and Simpson’s index of intestinal flora in mice in the DN group were all reduced, indicating that the diversity and abundance of the intestinal flora of DSS-induced ulcerative colitis mice were significantly reduced. The various alpha diversity indices of the intestinal flora of the mice were restored to different degrees after treatment with *Pediococcus pentosaceus* M6, which improved the intestinal flora disorders induced by ulcerative colitis (Figure 8B–D).

#### 3.8.2. Structure and Differential Analysis of Fecal Flora

**Figure 9:**
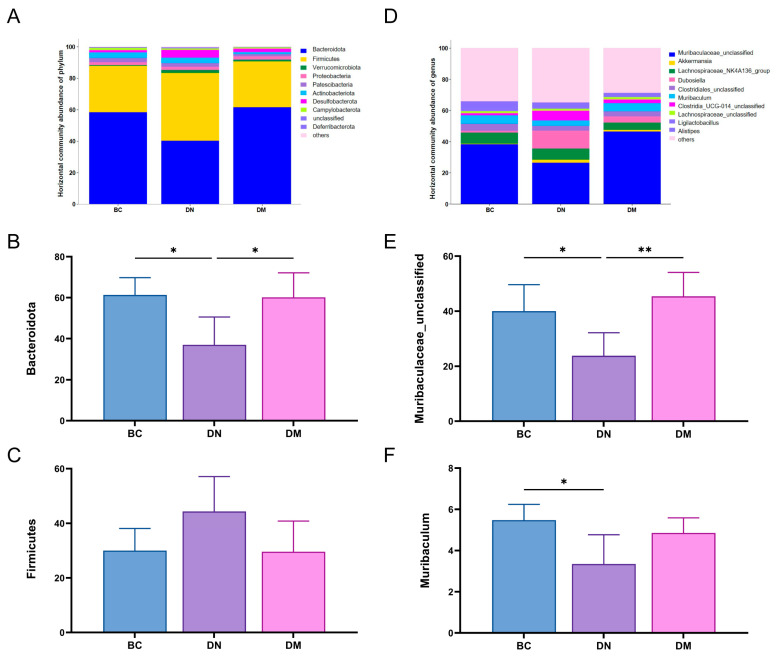
Effects of *Pediococcus pentosaceus* M6 on the structure and differences of intestinal flora in the mice with DSS-induced colitis. Note: * indicates *p* < 0.05; ** indicates *p* < 0.01. (**A**) The relative abundance of bacterial phyla at the phylum level across different groups; (**B**) the abundance of Bacteroidetes; (**C**) the abundance of Firmicutes; (**D**) the relative abundance of bacterial genera at the genus level across different groups; (**E**) the abundance of *Muribaculaceae*_unclassified; (**F**) the abundance of *Muribaculum*.

The results are shown in Figure 9. At the phylum level, the relative abundance of Bacteroides bacteria in the DN group decreased (*p* < 0.05) and the relative abundance of Firmicutes bacteria increased compared with the BC group, while the relative abundance of Bacteroides bacteria in the DM group increased (*p* < 0.05) and the relative abundance of Firmicutes decreased in the DM group compared with the DN group. At the genus level, compared to the BC group, the DN group showed a significant reduction in the relative abundance of *Muribaculaceae*_unclassified (*p* < 0.05) and *Muribaculum*. In contrast, the DM group had a significantly increased relative abundance of *Muribaculaceae*_unclassified compared to the DN group (*p* < 0.01), and also an increased relative abundance of the *Muribaculum* genus, although this increase was not statistically significant. The results indicated that *Pediococcus pentosaceus* M6 was able to regulate the disproportionate intestinal flora in the mice with ulcerative colitis. The results indicated that *Pediococcus pentosaceus* M6 could increase the proportion of probiotics, promote the restoration of intestinal flora, and maintain intestinal homeostasis.

## 4. Discussion

With the thriving development of probiotics and the probiotics industry, the development of targeted novel probiotics has become one of the critical research areas for advancing this industry. As a type of probiotics, lactic acid bacteria play a significant role, and it is crucial to screen for new lactic acid bacterial strains with probiotic properties from nature. Animal-derived probiotics play an important role in enhancing animal immunity, improving feed digestion and absorption, and preventing and treating diseases. Mongolian horses, as one of China’s ancient and superior local horse breeds, possess numerous excellent qualities. However, equine colitis is a common disease and cause of death among equines, and its high mortality rate has caused severe economic losses to the equine breeding industry. Therefore, in this study, we chose to screen for excellent lactic acid bacterial strains from the guts of Mongolian horses, providing the potential for producing probiotics to treat digestive diseases in equine animals.

### 4.1. Screening and Characterization of Strains

Lactic acid bacteria can be screened by changing the color of the indicator bromocresol purple, which has a pH color change range of 5.2 (yellow) to 6.8 (violet) [20]. In the experiment, an MRS medium containing bromocresol was used for cultivation, and colonies with a yellowish hue were selected to confirm their identity as lactic acid bacteria. A total of 96 lactic acid bacterial strains were examined in this study. Further screening was performed using the litmus milk test, in which litmus serves as both a pH indicator and an oxidation–reduction indicator, with a color change range of 4.5 (red) to 8.3 (blue). When prepared as a medium with a pH of 6.8, it appears bluish-purple. The main components of milk are lactose, casein, lactalbumin, and lactoglobulin. The fermentation of lactose, which produces large amounts of acid, can lead to the precipitation of casein, which is the main cause of milk curdling. In addition, some bacteria contain rennet, which can convert casein into paracasein [21]. Bacteria that contain lipase can break down the fat in milk and turn it into a yellow, transparent liquid. Bacteria containing caseinolytic enzymes can catalyze the hydrolysis of casein in milk and eventually turn it into a transparent liquid with caseinogen precipitate [22]. Lactic acid bacteria produce large amounts of acid during metabolism, which can also lead to the aggregation of casein in milk. Therefore, strains with pink curdled milk were selected in the experiment to identify them as lactic acid bacteria with good acidifying properties. Based on the results of the litmus milk test, 10 strains with good pink coagulation were selected from the 96 strains originally tested. The ability to produce acid is an important indicator for the evaluation of strains. Based on their acid production, strain M6, which showed the best acid production performance, was selected for further investigation.

There are many methods for classifying and identifying lactic acid bacteria. Traditional identification methods rely primarily on colony morphology, cell morphology, and the physiological and biochemical characteristics of the strains studied [23,24]. However, this method has a long experimental duration, and the experimental results are too dependent on the subjective judgment of the experimenter, making it impossible to specifically classify lactic acid bacteria. This method does not meet the current requirements for fast, accurate, and high-throughput identification of bacterial species. In recent years, molecular biology has been used to gradually establish molecular biological identification methods, in particular the 16S rDNA gene sequence analysis method, which has become an important method for the identification of lactic acid bacterial species. This method primarily uses the PCR technology to obtain the 16S rDNA gene sequence of the lactic acid bacteria to be identified, followed by sequencing to obtain genetic sequence information. The sequence obtained is then compared with strain sequences in the NCBI database, and the phylogenetic tree of the strain is constructed to determine its position in the tree and thus identify the lactic acid bacterial species to which the strain belongs [25].

In this study, the M6 strain was initially identified as a Gram-positive coccus based on colony morphology and microscopic examination results. Further identification was based on the physiological and biochemical characteristics of the lactic acid bacterial M6 strain with reference to the “Lactic Acid Bacteria Identification Manual” and the “Bergey’s Manual of Determinative Bacteriology”, whereby the investigated strain was provisionally identified as *Lactococcus*. Finally, strain M6 was definitively identified as *Pediococcus pentosaceus* based on the sequence analysis of the strain’s 16S rDNA gene.

### 4.2. Biofunctional Studies of Pediococcus pentosaceus M6

The growth curve is an important indicator for the evaluation of high-quality lactic acid bacteria. As candidate strains for microecological preparations, the rapid growth of lactic acid bacteria can make them the dominant flora in the intestinal tract and inhibit the growth of harmful bacteria [26]. A study showed that lactic acid bacteria can multiply rapidly in the initial phase of fermentation and play an important role in lactic acid fermentation [27]. The research results of Hwanhlem et al. [28] indicate that the logarithmic growth phase of lactic acid bacteria usually occurs 12 to 24 h after inoculation. Shao et al. [29] found that some strains entered the logarithmic growth phase at about 4 h, while others gradually entered the logarithmic growth phase at 6 h, and all of them reached a steady state of growth at 16 h. In this study, the logarithmic growth phase of the *Pediococcus pentosaceus* M6 was between 2~16 h, which could be due to differences between the strains studied. In order for lactic acid bacteria to colonize and function in the intestinal tract, they must tolerate the acidic environment of gastric juice. The survivability of the strains in the intestinal tract can be measured by their tolerance to bile salts [30]. The pH of equine gastric juice is between 1.0 and 7.0, and the transit time of food through the horse’s stomach is normally 30 min [31]. The survival rate of the M6 strain after a 30 min treatment in the artificial gastric juice with a pH of 3.0 was 4.77 ± 0.58%, and its survival rate in the intestinal fluid with a pH of 6.8 for 3 h was 8.99 ± 0.47%. Although its viability was significantly reduced in harsh gastrointestinal conditions, over 10^7^ CFU/mL of strain M6 remained viable, indicating it retains biological activity. Tolerance to acid and bile salts is an important indicator to measure whether a strain can colonize the intestinal tract. The research results of Reuben et al. showed that all the 15 lactic acid bacterial isolates tested had good tolerance to 0.3% bile salts after 6 h of exposure; only 6 strains of lactic acid bacteria could survive at a pH of 2.0 [32]. In the bile salt tolerance test of this study, the growth of *Pediococcus pentosaceus* M6 was not inhibited by bile salts at a concentration of 0.1%. However, when the bile salt concentration was 0.2% and 0.3%, the growth of *Pediococcus pentosaceus* M6 was inhibited and slowed down. *Pediococcus pentosaceus* M6 grew normally in the medium with a pH of 5.8 and was basically unaffected and grew normally in the medium with a pH of 4.0. In the medium with a pH of 3.0, the growth of *Pediococcus pentosaceus* M6 was relatively slow, and in the medium with a pH of 2.0, *Pediococcus pentosaceu*s M6 stopped growing or had stagnated growth. Therefore, it was determined that the *Pediococcus pentosaceus* M6 strain has good acid tolerance, can tolerate a low amount of bile salts, and has good adaptability to the intestinal environment of horses. The internal organ temperature of equine animals is below 40 °C, while the survival rate of *Pediococcus pentosaceus* M6 strain at 45 °C was 96.69 ± 2.20% and 60.69 ± 3.26% at 55 °C, indicating that the *Pediococcus pentosaceus* M6 strain can tolerate high temperatures, which is basically consistent with the research results of Khusro on lactic acid bacteria [33]. *Pediococcus pentosaceus* M6 in this study exhibited good biological characteristics.

### 4.3. Probiotic Effects of Pediococcus pentosaceus M6

The use of antibiotics in animal husbandry can lead to drug resistance. With the comprehensive ban on antibiotics in the livestock industry, there is a need to find new antibiotic alternatives, and lactic acid bacteria have received widespread attention. Firstly, the safety of lactic acid bacteria needs to be evaluated. *Pediococcus pentosaceus* M6 is a safe strain that does not produce hemolysis rings in hemolysis experiments and can be used as a potential probiotic. Secondly, the antibacterial and antibiotic resistance properties of lactic acid bacteria need to be evaluated. Several studies have shown that lactic acid bacteria isolated from different places have inhibitory effects on *Escherichia coli*, *Staphylococcus aureus*, and *Salmonella* [34,35]. The *Pediococcus pentosaceus* M6 strain screened in this study had good antibacterial effects on *Escherichia coli*, *Salmonella*, and *Staphylococcus aureus*, with the strongest inhibitory effect on *Salmonella* and the largest diameter of the inhibition zone of 16.71 ± 0.67 mm. Candidate strains for probiotics must be evaluated for their antibiotic sensitivity. The *Pediococcus pentosaceus* M6 strain screened in this study had different sensitivities to 30 antibiotics. It did not produce inhibition zones for 12 drugs, including cephalexin, ceftazidime, amikacin, gentamicin, kanamycin, neomycin, minocycline, norfloxacin, ciprofloxacin, vancomycin, Polyangiumycin, and cotrimoxazole, indicating that *Pediococcus pentosaceus* M6 was not sensitive to these 12 drugs; it had weaker sensitivity to four drugs, such as cefradine; it had strong sensitivity to nine antibiotics, such as ampicillin; and it had the strongest sensitivity to five antibiotics, such as penicillin. The results of this experiment were basically consistent with the previous experimental results, but there were also differences due to different strain sources and antibiotics [36,37].

### 4.4. Analysis of the Therapeutic Results of M6 Strains in DSS-Induced Colitis in Mice

Histopathology is recognized as one of the gold standards for diagnosing colitis [38]. Colon length and the disease activity index (DAI) are commonly used in clinical diagnosis to assess the severity of ulcerative colitis [39]. In the experiments, when the mice were given water containing dextran sulfate sodium (DSS), their DAI significantly increased, and H&E staining of colon tissue sections revealed a large number of inflammatory cells in the colonic mucosa and submucosa of the mice, indicating that DSS successfully induced a mouse model of ulcerative colitis. After treatment with *Pediococcus pentosaceus* M6, the DAI of the mice decreased, the colon length approached that of the baseline control (BC) group, and H&E staining of colon sections showed a significant reduction in inflammatory cell infiltration. These results suggest that *Pediococcus pentosaceus* M6 can alleviate DSS-induced colitis in mice in both clinical indicators and pathological changes.

Cytokines are active substances that mediate immunity and inflammation [15]. They play crucial regulatory roles in cell growth, differentiation, and intercellular interactions and have significant effects on immune–inflammatory responses [40]. Colitis is characterized by abnormal levels of inflammatory cytokines, and numerous studies have confirmed that the levels of inflammatory cytokines are associated with the pathological progression of colitis [41]. Cytokines are divided into pro-inflammatory cytokines and anti-inflammatory cytokines, with IL-6, IL-1β, and TNF-α being pro-inflammatory cytokines and IL-10 being a commonly used anti-inflammatory cytokine. Pro-inflammatory cytokines IL-6 and IL-1β primarily increase intestinal permeability by generating macrophages in colonic tissue [42,43]. Interleukin 6 and IL-1β play vital roles in the pathophysiology of inflammatory diseases by accelerating neutrophil recruitment and inhibiting T cell differentiation, which are used to monitor the occurrence and development of colitis. Studies have shown that DSS-induced colitis in mice results in increased levels of IL-6 and IL-1β [44], consistent with the findings of this study. IL-10 plays a crucial immunoinhibitory role in certain diseases by promoting B cell proliferation, maintaining suppressive functions, and accelerating antibody secretion. The results of this study indicate that *Pediococcus pentosaceus* M6 can reduce the expression of IL-6 and IL-1β and increase the expression of IL-10, suggesting that *Pediococcus pentosaceus* M6 can effectively alleviate the progression of colitis in mice.

The intestinal barrier is the foundation for regulating intestinal homeostasis and the first line of defense for the body against pathogen invasion [45]. The intestinal barrier is a complex structure that plays a crucial role in the host’s response to intestinal pathogens and the intestinal microbiota. Evidence has shown that disruption of the intestinal barrier can lead to severe tissue damage and microbial invasion [46]. Therefore, the protection and repair of the intestinal barrier are essential for preventing intestinal microbial invasion and reducing inflammatory responses. Tight junction proteins are components of the colonic mechanical barrier that prevent the dissemination of microbial toxins and antigens. The reduction of tight junction proteins indicates intestinal barrier dysfunction. Therefore, this study measured the expression of tight junction protein-related genes *ZO-1* and *occludin* [47]. Zonula occludens-1 was the first epithelial tight junction protein to be reported [48]. Proteins such as ZO-1 within the cell are signaling molecules that connect the tight junction protein complex to the actin cytoskeleton [49]. Kuo et al. [48] found that the absence of ZO-1 increases intestinal barrier permeability and exacerbates the development of colitis. The absence of *occludin* is thought to lead to the disruption of colonic epithelial connections [50]. Therefore, preventing the loss of tight junction proteins is an important therapeutic approach for maintaining the intestinal barrier function. In this study, after successful DSS induction, the mRNA expression levels of tight junction proteins *ZO-1* and *occludin* decreased, consistent with previous studies [44]. After intervention with the *Pediococcus pentosaceus* M6, the mRNA expression levels of tight junction proteins ZO-1 and *occludin* increased. This suggests that *Pediococcus pentosaceus* M6 effectively attenuates the reduction of tight junction proteins in DSS-induced colitis in mice and alleviates the degree of intestinal barrier damage in DSS-induced colitis in mice.

### 4.5. Effects of M6 Strain on Gut Microbiota in DSS-Induced Colitis Mice

The gut microbiota is composed of tens of thousands of symbiotic microorganisms, which play a crucial role in maintaining the intestinal barrier function, immune system development, and immune function regulation [51]. A growing body of research has confirmed that an imbalance in the gut microbiota is considered a cause of the onset and progression of colitis [52]. A clinical study revealed that there are differences in the gut microbiota structure between colitis patients and healthy individuals, particularly in terms of microbial diversity and the proportions of specific bacterial taxa [53]. For instance, compared to healthy individuals, the diversity of the gut microbiota in colitis patients is significantly reduced [54]. At the phylum level, Bacteroidetes play a pivotal role in carbohydrate hydrolysis, polysaccharide metabolism, and the metabolism of bile acids and steroids. Additionally, Bacteroidetes can interact with cellular receptors through lipopolysaccharides and flagellin components, enhancing immune responses via cytokine synthesis [55]. The Firmicutes phylum has been shown to be closely associated with inflammatory processes [56]. Induction by DSS leads to an increase in the Firmicutes/Bacteroidetes ratio, which has been utilized as a biomarker for various pathological conditions, including intestinal inflammation [57]. This experiment yielded similar results, showing an elevated Firmicutes/Bacteroidetes ratio in the DN group compared to the BC group. The DM group exhibited a reduction in the Firmicutes/Bacteroidetes ratio, indicating that the treatment in the DM group had a beneficial regulatory effect on the gut microbiota, alleviating the DSS-induced increase in the Firmicutes/Bacteroidetes ratio. This suggests that the M6 strain possesses the potential to reverse gut microbiota dysbiosis. At the genus level, studies have shown that *Muribaculaceae*_unclassified is a beneficial bacterial genus in the mouse gut. As a member of the *Muribaculaceae* family, *Muribaculaceae*_unclassified is believed to play an important role in regulating host health by degrading mucin [58,59]. It has been reported in animal studies that, compared to the control group, the addition of probiotics upregulates beneficial microbiota and downregulates or leaves unchanged the population of *Clostridium*, indicating that probiotics can modulate the structural composition of the gut microbiota [60]. In the results of this experiment, the DM group upregulated *Muribaculaceae*_unclassified and *Muribaculum*, which is consistent with these findings, suggesting that the M6 strain treatment group increased the proportion of beneficial bacterial genera.

## 5. Conclusions

The aforementioned results indicate that *Pediococcus pentosaceus* M6, isolated and screened from the intestinal contents of Mongolian horses, exhibits excellent probiotic properties and potential as a probiotic. Furthermore, through a meticulous study using a mouse model, we discovered that *Pediococcus pentosaceus* M6 has the potential to treat ulcerative colitis in mice, providing a reference for future research on the use of probiotics in studying gastrointestinal diseases in equines.

## Figures and Tables

**Table 1:** Physiological and biochemical identification form.

Project	Result	Project	Result
Esculin hydrate	−	Power	+
D(+)-cellobiose	+	H_2_S	−
Maltose	+	Indole	−
Mannitol	+	H_2_O_2_	−
D(−)-salicin	+	Gelatin starch hydrolysis	+
Sorbitol	−	Nitrate	+
Sucrose	+	V-p	−
Raffinose	+	Mr	+
Inulin	−	Glucose gas production	+
Lactose	−	1% sodium hippuric acid	−

Note: “+” indicates positive, fermentation of this substrate, or presence of this phenomenon; “−” indicates negative, no fermentation of this substrate, or absence of this phenomenon.

**Table 2:** Statistical table of simulated gastrointestinal fluid test results.

Time, h	Gastric Fluid Survival Rate, %	Intestinal Fluid Survival Rate, %
0	100	100
3	8.99 ± 0.47	4.77 ± 0.58

**Table 3:** Bacteriostatic ability of the M6 strain, means ± SD.

Pathogenic Bacteria	Inhibitory Circle Diameter (mm)
*Escherichia coli*	9.50 ± 0.69
*Salmonella*	16.71 ± 0.67
*Staphylococcus aureus*	14.29 ± 0.69

Note: the size of the inhibitory ability of the strain is the same as the size of the inhibitory circle. The diameter of the inhibitory circle of 0~10 mm indicates a good inhibitory effect, and the diameter of the inhibitory circle of 10~20 mm indicates an excellent inhibitory effect.

**Table 4:** Antibiotic susceptibility results.

Antibiotics	Sensitivities	Antibiotics	Sensitivities
Penicillin	+++	Neomycin	−
Oxacillin	+	Achromycin	++
Carbenicillin	+++	Deoxytetracycline	++
Ampicillin	++	Minocycline	−
Piperacillin	++	Erythromycin	++
Cefalexin	−	Midecamycin	+++
Cefazolin	+	Norfloxacin fluoride	−
Cefradine	+	Norfloxacin	−
Cefuroxime	+	Ciprofloxacin	−
Ceftazidime	++	Vancomycin	−
Ceftriaxone	−	Polyglycosamicin	−
Cefoperazone	++	Bactrim	−
Butylamine kaner	++	Foroxone	++
Gentamicin	−	Clindamycin	++
Kanamycin	−	Amchloromycin	+++

Note: Diameter of the inhibition circle (mm): +++, 20~30 mm; ++, 10~29 mm; +, 1~9 mm; −, no inhibition circle.

## Data Availability

The original contributions presented in the study are included in the article/Supplementary Materials. Further inquiries can be directed to the corresponding author.

## Supplementary Materials

The following supporting information can be downloaded at: https://www.mdpi.com/article/10.3390/microorganisms13050957/s1, Figure S1: Results of screening and identification of equine-derived strains; Table S1: Scoring system for the disease activity index (DAI); Table S2: histopathological scoring standard; Table S3: teverse transcription reaction system; Table S4: primer information; Table S5: real-time fluorescence quantitative PCR reaction system.
