# Supplementary material for: Probiotic Potential of Pediococcus pentosaceus M6 Isolated from Equines and Its Alleviating Effect on DSS-Induced Colitis in Mice
Source: Microorganisms. 2025 Apr 22;13(5):957. doi: 10.3390/microorganisms13050957 (PMC12114451; doi:10.3390/microorganisms13050957)
Supplement: Supplementary file 1 [file microorganisms-13-00957-s001.zip › Figure S1. Results of screening and identification of equus-derived strain..pdf]

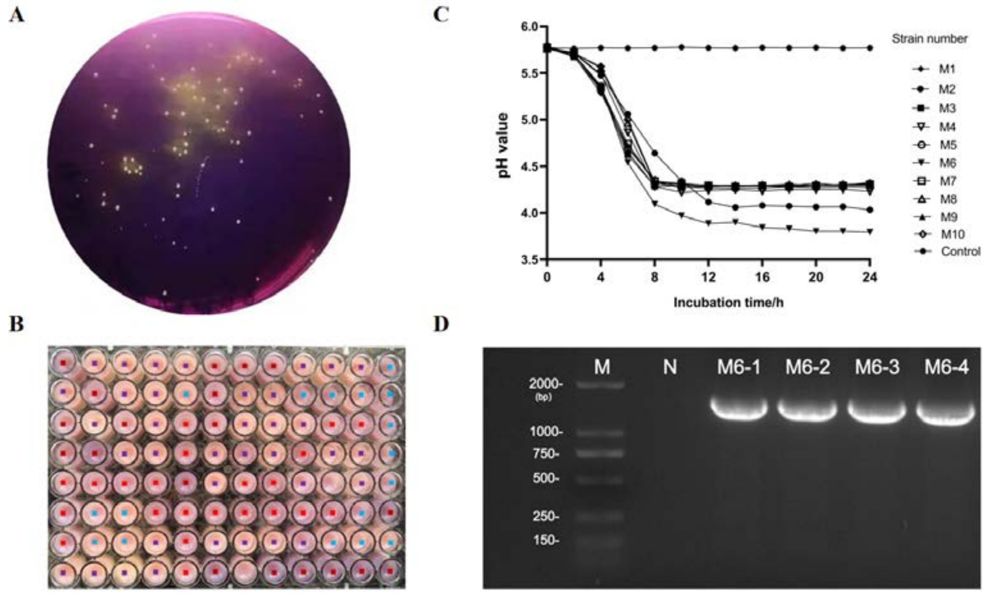

Figure S1. Results of screening and identification of equus-derived strain. (A) Bromocresol Purple Color test results; (B) Litmus milk color test results, Orange markers indicate that color development has not occurred without curd, purple markers pink with curd, blue markers pink without curd, and red markers with curd with weak color development; (C) Acid production curve; (D) Agarose electrophoresis picture of M6 strain,PCR amplified fragments of M.DL2,000DNA Marker1-3, N are blank controls, M6-1 is the amplification product of strain M6, and M6-2, M6-3, and M6-4 are parallel controls.
